# Predicting castration-resistant prostate cancer after combined androgen blockade

**DOI:** 10.18632/oncotarget.22246

**Published:** 2017-11-01

**Authors:** Miao He, Haina Liu, Jingyi Cao, Qian Wang, Haiting Xu, Yufeng Wang

**Affiliations:** ^1^ Department of Nuclear Medicine, Xuzhou Cancer Hospital, Jiangsu Province, Xuzhou, China; ^2^ Department of Urology, Xuzhou Cancer Hospital, Jiangsu Province, Xuzhou, China; ^3^ Department of Radiotherapy and Oncology, Xuzhou Cancer Hospital, Jiangsu Province, Xuzhou, China

**Keywords:** prostate cancer, bone metastasis, bone scan, combined androgen blockade, castration resistance

## Abstract

This study analyzed ^99^Tcm-MDP bone scans and investigated factors influencing early-stage castration resistance in prostate cancer (CRPC) patients with bone metastasis. We retrospectively analyzed clinical data from 92 patients with bone metastatic prostate cancer treated with maximal androgen blockade. Patients were imaged with ^99^Tcm-MDP bone scan to detect metastases, and prostate specific antigen (PSA) values were measured regularly. Before treatment, 464 total bone metastases were detected in the 92 patients, with pelvic bone metastases accounting for about 30.6% of the total. After combined androgen blockade treatment, median CRPC occurrence time was 23 months. A longer time to reach the lowest PSA value was an independent predictor of early-onset CRPC (occurrence <1 year after treatment). Our findings suggest that ^99^Tcm-MDP bone scans are useful for diagnosing prostate cancer bone metastasis and grading. Patients with Gleason scores>8, higher PSA values after treatment, and shorter times to reach the lowest PSA value had poorer responses to combined androgen blockade treatment.

## INTRODUCTION

Prostate cancer incidence differs depending on geographical location and patient ethnicity. In Europe, the United States, and other developed nations, prostate cancer is the most common malignancy in males [1]. While prostate cancer incidence is lower in Asian than Western countries, prostate cancer has become increasingly common in Asia. Prostate cancer is now the third most common malignancy of the male urinary tract and reproductive system in China, seriously affecting quality of life in elderly men.

More than 90% of prostate cancer patients will eventually have bone metastases. Early diagnosis of prostate cancer with or without bone metastases, and accurate assessment of bone metastasis locations and quantities are very important for therapeutic decision-making. ^99^Tcm-MDP is a noninvasive, highly sensitive radiotracer commonly used to screen for malignant tumors with or without bone metastases. 99Tcm-MDP is frequently used to diagnose prostate cancer patient bone metastases and to evaluate therapeutic efficacies [2].

Prostate cancer with bone metastases is an advanced stage disease with considerably poorer prognosis. Combined androgen blockade therapy is often the preferred treatment for these patients [3]. We followed-up patients whose bone metastases were imaged via single-photon emission computerized tomography (SPECT), and found that nearly all of these patients experienced disease progression and developed castration resistant prostate cancer (CRPC) [4]. More accurate clinical indicators and biomarkers are needed to inform personalized treatment options for patients with CRPC.

This study retrospectively analyzed prostate cancer patient data to identify prostate cancer bone metastasis clinical features and evaluate the effectiveness of combined androgen blockade therapy in patients with bone metastases. We assessed strategies for early identification of patients with bone metastases insensitive to combined androgen blockade therapy, and explored personalized treatment regimens for metastatic prostate cancer.

## RESULTS

In this study, 92 prostate cancer cases were diagnosed with bone metastases and were ^99^Tcm-MDP bone scan positive. According to the Soloway grading criteria, 36/92 prostate cancer cases were diagnosed via ^99^Tcm-MDP bone scans as grade I, 24/92 as grade II, and 32/92 as grade III. The total number of bone metastases diagnosed using ^99^Tcm-MDP was 464, with 30.6% found in the pelvic bone, 23.7% in the spine, 19.2% in ribs, and 12.5% in limbs (most commonly in the proximal femur) (Table 1).

**Table 1:** Distribution of bone metastases

Site	Number of bone metastases (%)
Pelvis	142 (30.6%)
Spine	110 (23.7%)
Ribs	89 (19.2%)
Limb bones	58 (12.5%)
Sternum	36 (7.8%)
Skull	16 (3.4%)
Others	13 (2.8%)

All 92 patients with bone metastases underwent anti-androgen therapy after initial SPECT bone scan, and were followed up regularly using bone scans and serum prostate specific antigen (PSA) measurements. Patient clinical characteristics are shown in Table 2. Receiver operating characteristic curve analysis (ROC) was used to determine whether the lowest PSA values and the time to reach the minimal PSA value could be used to predict CRPC. The results showed that the optimal cutoff value of the lowest PSA values and the time to reach the minimal PSA value for predicting CRPC were 0.2 ng/mL and 9 months, respectively. CRPC was diagnosed in 51 patients during the follow-up period. Early CRPR (development within one year of treatment) was diagnosed in 24 patients (47%). Mean time to CRPC was 23 months. Patients were grouped according to clinical indicators, and chi-square test results showed that patients with Gleason scores (8–10vs. 6–7) had the lowest PSA values after combined androgen blockade therapy, and the time to reach the minimal PSA value was associated with CRPC development after treatment (Table 2).

**Table 2:** Relationships between clinicopathological characteristics and CRPC in patients with prostate cancer

Characteristics	Group	Total number of patients	Number of patients with CRPC	*P*-value
Age	≤75	50	31	0.21
	>75	42	20	
Preoperative PSA (ng/mL)	≤100	45	22	0.21
	>100	47	29	
Gleason score	<8	67	30	<0.01
	≥8	25	21	
Number of bone metastases	≤4	43	23	0.83
	>4	49	28	
Lowest value of PSA after maximal androgen blockade therapy (ng/mL)	≤0.2	50	14	<0.01
	>0.2	42	37	
Time to reach minimal PSA (months)	≤9	52	36	<0.01
	>9	40	15	

According to non-parametric and chi-square analyses, early-onset CRPC patients had the highest PSA values, and the longest times to reach the lowest PSA values (Table 3). Logistic regression analysis showed that time to lowest PSA value was an independent predictor of early-onset CRPC (Table 4). We found that PSA value decline patterns in patients who underwent combined androgen blockade therapy could indicate CRPC occurrence. CRPC developed earliest in patients with the highest PSA values (≥0.2 ng/mL) and the longest times to the lowestPSA values (Table 3).

**Table 3:** Relationships between clinicopathological characteristics and CRPC in patients with prostate cancer after one year of treatment

Variable	Median time to CRPC			*P*-value
		≥1 year	<1 year	
Preoperative PSA (ng/mL)		109	123	0.27
Gleason score	<8	19	13	0.26
	≥8	8	11	
Number of bone metastases	≤4	15	10	0.40
	>4	12	14	
Lowest PSA value after maximal androgen blockade therapy (ng/mL)		0.4	1.92	<0.01
Time to reach minimal PSA (months)		8.5	3.9	<0.01

**Table 4:** Multivariate analysis in patients with bone metastases who underwent anti-androgen therapy for CRPC after one year of initial treatment

Variable	Group	HR	95% CI	*P-*value
Lowest PSA value after maximal androgen blockade therapy	≤0.2	1		
	>0.2	21.6	6.4–73.0	<0.01
Time to reach minimal PSA (months)	≤9	1		
	>9	0.2	0.1–0.7	<0.01

HR: hazard ratio; CI: confidence interval.

## DISCUSSION

Metastasis to the bone is a common problem in prostate cancer. Tumor cells reach bone sites mainly via inferior vena cava blood reflux, followed by arterial flow to the body, through the deep vein of the penis to the Batson vertebral venous plexus, finally reaching the pelvis and lumbar vertebrae [5, 6]. Batson suggested that there is a “portal-like” vein system between the prostate and the low lumbar spine, leading to increased risk of prostate cancer metastasis to the spine. Prostate cancer can also metastasize to the pelvis [7, 8]. In this study, 464 bone metastatic lesions were diagnosed via ^99^Tcm-MDP bone scan, the majority of which were found in the pelvic bone (30.6%) or spine (23.7%).

The most commonly used first-line clinical treatment for prostate cancer patients with bone metastases is combined androgen blockade, which is more efficacious than castration or anti-androgen therapy alone [9]. However, almost all patients experience disease progression (CRPC) at some point after initial treatment. For those patients insensitive to combined androgen blockade treatment, more sensitive predictors of disease progression will allow for better therapeutic decision-making and patient outcomes. In this retrospective study, we found that Gleason scores and PSA changes after combined androgen blockade therapy were associated with treatment outcomes. Patients with high Gleason scores, high minimum PSA values, and short times to reach minimum PSA values after treatment developed CRPC earlier than other patients, and these factors independently predicted early-onset CRPC.

Gleason score is an important parameter in prostate cancer grading. High Gleason scores represent high tumor heterogeneity and poor tumor cell differentiation, indicating loss of glandular structure. Our study showed that prostate cancer patients with Gleason scores of 8–10 had were at higher risk for CRPC occurrence after maximal androgen blockade. Combined androgen blockade treatment was not efficacious in these patients, and early combined treatment should be considered.

Measurement of PSA in serum is an excellent method for early detection and diagnosis of prostate cancer [10]. At present, PSA is the first line method for prostate cancer monitoring [11, 12]. Our study showed that there was no relationship between PSA level before combined androgen blockade treatment and time to CRPC after treatment (P>0.05), although changes in PSA were directly associated with time to CRPC. Patients with the lowest PSA values developed CRPC later than other patients, and this was a good marker for combined androgen blockade treatment efficacy. Combined androgen blockade reduced PSA values in most patients by more than 80% in the first month [13]. However, some studies had shown that PSA decline did not indicate tumor cell death, but instead represented AR pathway inhibition and subsequent reduced PSA secretion [14]. However, a slower PSA reduction following an initial rapid decline is considered representative of tumor load reduction. Thus, we analyzed the relationship between the time to PSA minimum value and CRPC incidence post-treatment. Our findings suggested that CRPC occurred later in patients whose PSA measurements reached minimum values>9 months post-treatment. Sasaki, *et al.* and Hussain, *et al.* similarly concluded that patients with longer times to minimum PSA values had longer overall survival times than those with shorter times to minimal PSA [15].

In summary, prostate cancer is highly susceptible to bone metastases. We found that patients with Gleason scores>8, PSA minimum values>0.2 ng/mL, and times to minimum PSA value<9 months had poor responses to maximal androgen blockade treatment. Therefore, early combined treatment should be considered in these patients.

## MATERIALS AND METHODS

### Subjects

This study retrospectively analyzed clinical data from 92 cases of prostate cancer with bone metastasis treated usingcastration combined with oral anti-androgen therapy between 2010 and 2015 at the Xuzhou Tumor Hospital. All patients underwent routine bone scans, rectal prostate ultrasound, PSA and free PSA assessments, and prostate biopsy before treatment to confirm the diagnosis of prostate cancer with bone metastasis. After castration surgery, patients began anti-androgen therapy with either bicalutamide or flutamide, and regular outpatient follow-up bone scans and PSA measurements were performed.

The 2014 European Union Urological Association guidelines define CRPC biochemically as three consecutive PSA increases (each one week apart), two PSA measurements >50% above the lowest value, and PSA >2 ng/mL. Early CRPC was defined as CRPC occurring within one year of combined androgen blockade therapy.

### Statistical analysis

Statistical analyses were performed using SPSS 16.0 software. P<0.05 was considered statistically significant. Nonparametric and chi-square tests were used to compare differencesbetween early CRPC and other CRPC patients. Logistic regression was used to analyze independent factors associated withearly CRPC.
